# Assessment of Homodyned K Distribution Modeling Ultrasonic Speckles from Scatterers with Varying Spatial Organizations

**DOI:** 10.1155/2017/8154780

**Published:** 2017-09-05

**Authors:** Xiao Hu, Yufeng Zhang, Li Deng, Guanghui Cai, Qinghui Zhang, Yi Zhou, Kexin Zhang, Junhua Zhang

**Affiliations:** ^1^Department of Electronic Engineering, Yunnan University, Kunming City, Yunnan Province, China; ^2^Cardiovascular Department, The Second Affiliated Hospital of Kunming Medical University, Kunming City, Yunnan Province, China

## Abstract

**Objective:**

This paper presents an assessment of physical meanings of parameter and goodness of fit for homodyned K (HK) distribution modeling ultrasonic speckles from scatterer distributions with wide-varying spatial organizations.

**Methods:**

A set of 3D scatterer phantoms based on gamma distributions is built to be implemented from the clustered to random to uniform scatterer distributions continuously. The model parameters are obtained by maximum likelihood estimation (MLE) from statistical histograms of the ultrasonic envelope data and then compared with those by the optimally fitting models chosen from three single distributions. Results show that the parameters of the HK distribution still present their respective physical meanings of independent contributions in the scatterer distributions. Moreover, the HK distribution presents better goodness of fit with a maximum relative MLE difference of 6.23% for random or clustered scatterers with a well-organized periodic structure. Experiments based on ultrasonic envelope data from common carotid arterial B-mode images of human subjects validate the modeling performance of HK distribution.

**Conclusion:**

We conclude that the HK model for ultrasonic speckles is a better choice for characterizing tissue with a wide variety of spatial organizations, especially the emphasis on the goodness of fit for the tissue in practical applications.

## 1. Introduction

The ultrasonic imaging has many advantages over other techniques due to utilizing nonionizing radiation, scanning in real time, and distinguishing soft tissues with high sensitivity and resolution [1, 2]. The speckle, which manifests the granular structure in the ultrasound images, is caused by diffuse scattering of the ultrasound, and the background texture of the speckle is connected with the tissue microstructure. Therefore, the ultrasound imaging shows good potential for diagnosing diseases by statistical analysis of the speckle properties in the images to extract corresponding distribution parameters [1, 2]. Two kinds of statistic models including single distributions [3–5], such as the K distribution (K), Rayleigh distribution (RA), Rician distribution (RI), and Nakagami distribution, as well as compound distributions [6–9], such as the homodyned K distribution (HK), generalized K distribution, Rician inverse Gaussian distribution (RiIG), Nakagami-generalized inverse Gaussian distribution (NGIGD), have been investigated for analyzing the statistical properties of the ultrasonic-echoed envelope data. As commonly used models, the single distributions have been widely employed since the 1980s [3–5]. This kind of method is used as the histological descriptors with a one-to-one relationship between the distribution type and tissue characterization. According to the results from the researches, the K distribution corresponds to the tissue with low density of scatterers and without a deterministic component; the Rayleigh distribution refers to the tissue with high density of scatterers and a without deterministic component; the Rician distribution represents tissue with high density of scatterers and a deterministic component. On the contrary, the compound distributions model the tissue speckle pattern in images through modulating the parameters to represent the scatterer clustering degree or effective density, diffuse signal power, and coherent signal component [6–9]. The quantitative measurements, such as log-likelihood cross-validation or Kullback-Leibler distance, are used to verify the model performance.

As a generalized compound distribution, the HK model has drawn more attention over the other compound versions because its parameters present respective physical meanings from independent contributions in the scatterer distributions. In order to investigate the parameter meaning of the HK distribution, Prager et al. [10] described a method to estimate the ratio of the mean to the standard deviation and the skewness for the statistical model of the HK distribution based on arbitrary powers of the simulating ultrasound echo envelope signals. The parameters of the HK distribution were also estimated by the moments of the distribution. As a unifying point of view, Destrempes and Cloutier [11] compared the HK distribution and other statistical models based on theoretical computation for the modulated distribution, modulating distribution, and modulated parameters on the mean and the signal-to-noise ratio of the signal intensity. The authors conclude that the HK distribution is the only model that the parameters have their physical meanings in certain cases, even though the other distributions may better fit ultrasound signals. In addition, the authors suggest that the goodness of fit for HK distribution should be further assessed by the simulation or clinical test. Destrempes et al. [12] presented a new estimation method for the parameters of HK distribution by the mean intensity and two Log-moments. Then, they made a comparison between this method and the methods based on the first three moments of the intensity, the amplitude, or the signal-to-noise ratio (SNR), skewness, and kurtosis of two fractional orders of amplitude. The results indicate that this estimation method is the best. However, the method of moments for parameter estimation is deficient because the solutions of the equations based on the even moments are not always real or positive. The selection criteria for a set of parameters are various and nonunique, and the computational complexity for the high order moments is also a problem [13]. Moreover, the distribution based on the moment method may not be the optimum one for fitting ultrasound signals. As an important aspect in practice, applications of the best fitting ultrasound signals using statistical models involve tissue segment [14], speckle reduction [15], modeling for localizing a thin surgical tool [16], ultrasound kidney images [17], carotid artery plaque assessment [18], or classification of breast lesions [19]. It is necessary to assess the parameter meanings and goodness of fit of HK distribution for ultrasound echo signals under an optimum condition.

The objective of this paper is to assess the physical meanings of parameter and goodness of fit of HK distribution for ultrasonic envelope data from scatterer distributions with wide-varying spatial organizations by using maximum likelihood estimation (MLE) criteria. A 3D scatterer phantom based on gamma distributions is built to be implemented from the clustered to random to uniform scatterer distributions continuously. The model parameters and maximum likelihood estimation are obtained by MLE from statistical histograms of the ultrasonic envelope data. In order to evaluate the parameter meanings and goodness of fit, the mean and standard deviation (MSD) of these estimated values based on 30 simulation realizations are compared with those based on the optimally fitting models chosen from commonly used three single distributions, that is, the K, Rayleigh, and Rician (OKRR) distributions. Experiments based on ultrasonic envelope images from common carotid arteries (CCA) of 30 human subjects validate the simulation results of HK distribution for tissues with varying scatterer spatial organizations.

## 2. Methods

### 2.1. The Speckle Models

#### 2.1.1. Three Single Distributions

(i) *Rayleigh Distribution*. The Rayleigh distribution [20] arises with a large number of scatterers in the effective resolution cell. The scattering structure is too fine to be resolved and fully forms a speckle pattern in ultrasonic B-mode images. The Rayleigh distribution for ultrasonic envelope amplitude *A* is defined by
  (1) $$\begin{array}{c} P_{RA}\left(A\right)=\frac{A}{d^{2}}e^{-A^{2}/2d^{2}}, \end{array}$$
  where *d* ^2^ represents the variance of scatterer strength. This distribution is a classical statistical model that assumes many fine randomly distributed scattering sites in the space without any well-organized periodic structure.
(ii) *Rician Distribution*. The Rician distribution [21] describes the analogous textures as the Rayleigh distribution, but the difference is the existence of the coherent signal echoed from the well-organized periodic scatterer structure to the diffuse signal from randomly distributed scatterers. The Rician distribution is expressed as
  (2) $$\begin{array}{c} P_{RI}\left(A\right)=\frac{A}{g^{2}}e^{-\left(A^{2}+l^{2}\right)/2g^{2}}I_{0}\left(\frac{Al}{g^{2}}\right), \end{array}$$
  where *g* ^2^ and *l*, respectively, are the variance and mean in scatterer strength. *I*_0_(*x*) is the modified Bessel function of the first kind and order zero. The special case is that the Rician distribution becomes the Rayleigh model with a small value of *l* or Gaussian model for *l* → ∞.
(iii) *K Distribution*. Another commonly used model for ultrasonic envelope data is called the K distribution [22], which may describe the signals from the structures with a small number of scatterers in the effective resolution cell. The probability density function for the envelope amplitude *A* can be written as
  (3) $$\begin{array}{c} P_{K}\left(A\right)=\frac{2a}{\Gamma \left(m\right)}{\left(\frac{aA}{2}\right)}^{m}K_{m-1}\left(aA\right), \end{array}$$
  where *K*_*m*−1_(*x*) is the modified Bessel function of the second kind and order *m* − 1. Γ(*x*) is the gamma function, and *a* = 2(*m*/(2*σ* ^2^))^1/2^ (where 2*σ* ^2^ is the second moment of *A*). For the case of *m* → ∞, this model turns into the Rayleigh distribution.

#### 2.1.2. The Homodyned K Distribution

The HK distribution [10], as a more universal statistical model, is used to describe the signals from the structure filling of variable density scatterers with or without well-organized periodic components. The HK distribution models the ultrasonic envelope amplitude *A* by

(4) $$\begin{array}{c} P_{HK}\left(A\right)=\int {AuJ}_{0}\left(u\varepsilon \right)J_{0}\left(uA\right){\left(1+\frac{u^{2}\sigma^{2}}{2c}\right)}^{-c}du, \end{array}$$

where *ε*, *σ*, and *c*, respectively, denote the coherent component, diffuse component, and scatterer clustering degree in the signal. *J*_0_(*x*) is the Bessel function of the first kind with order 0. The model shades into the K distribution with *ε* → 0, the Rayleigh distribution with *c* → ∞ and *ε* → 0, and the Rician distribution with *c* → ∞. The parameters of the HK distribution have their own physical meanings of the independent contributions from clustered, random, and regular components in the scatterer distributions.

### 2.2. Ultrasound-Echoed Data Simulation

In order to objectively and fully assess the HK distribution performance, it is required to synthesize a varied ultrasonic data source with the scatterer distributions, whose density and spatial organization can be tuned along the continuum from clustering to random to regular. In a present study, a 3D simulation for the ultrasonic envelope images is performed by the Field II software on the MATLAB platform. This library achieves this target by setting the scatterer phantom geometry, density, strength, and organization as well as the probe and ultrasonic scanning parameters in relevant functions.

**Figure 1:**
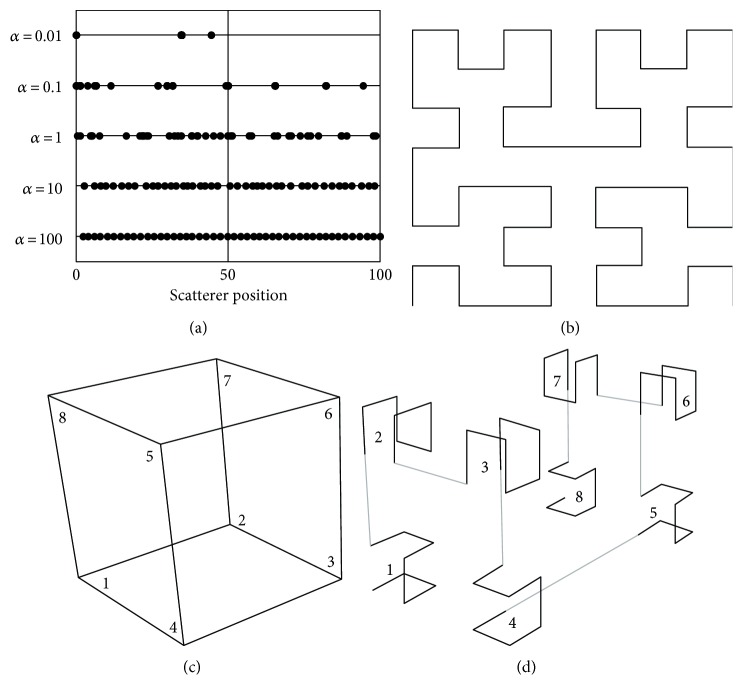
The scatterer position by the gamma distributions with different shape parameters *α* in one dimension (a), and mapping schematic to two dimensions (b), three dimensions with levels 1 (c) and 2 (d).

A generalized Poisson process is used to setup the 3D scatterer distribution by a given scatterer number and phantom dimension as well as shape and scale parameters of the gamma distribution for the scatterer space. A one-dimensional scatterer model proposed by Cramblitt and Parker [23] is given by

(5) $$\begin{array}{c} s\left(x\right)=\sum_{i}a_{i}\delta \left(x-X_{i}\right), \end{array}$$

where *X*_*i*_ and *a*_*i*_ are position and strength of the *i*th scatterer, respectively. A Poisson process is considered to define the distance *d* between two continuous points [24]. In this case, the space of scatterers is the gamma distribution to generalize this Poisson process with the shape parameter *α* and scale parameter *β* as

(6) $$\begin{array}{c} f\left(d\right)=\frac{d^{\alpha -1}\exp\left(-d/\beta \right)}{\Gamma \left(\alpha \right)\beta^{\alpha }}, \end{array}$$

where *α* > 0, *β* > 0, and *d* > 0. The mean and variance of the space *d* are $\bar{d}=\alpha \beta$ and $\sigma_{d}^{2}={\alpha \beta }^{2}={\bar{d}}^{2}/\alpha$, respectively. Therefore, the scatterer distribution could be characterized by the density parameter $1/\bar{d}$ and shape parameter *α*. For *α* < 1, the scatterer distribution is clustering with high space variance; with *α* = 1, the Poisson process with gamma distribution turns into exponential distribution, and the space *d* is random; for *α* > 1, the scatterers are distributed evenly in the space with low space variance. In other words, *d* is set to equality.  Figure 1(a) demonstrates the one-dimensional scatterer positions with different values of shape parameter *α* under a certain density condition (determined by $\bar{d}$). The shape parameter *α* is set as 0.01, 0.1, 1, 10, and 100, while the scale parameter *β* changes with the *α* by $\beta =\bar{d}/\alpha \;\left(\bar{d}=2.17\right)$ with 50 scatterers. In this figure, the clustering degree of scatterers is the highest for *α* = 0.01. With the value of the shape parameter increasing, the clustering degree is decreasing, and the scatterer positions are randomly distributed with *α* = 1, while the distribution tends to evenly spread as *α* = 100. Therefore, with the shape parameter increasing, the scatterer distribution is changing from clustered to random to regular continuously. By the given varied mean distance (density) and shape parameter, the scatterer distance distribution could be smoothly changed from irregularity to regularity, which makes this scatterer model agilely and continuously adjustable.

For resembling the reality, the one-dimensional scatterer distribution should be isotropically mapped onto two- or three-dimensional spaces for guaranteeing homogeneity to the scatterer structure. As a continuous and nondifferentiable fractal, space-filling Hilbert curve [25] becomes a good choice because it could well assure the corresponding relation between the distance for two contiguous points of the original one-dimension line and the spatial distance for two points of the multidimension space. For the 2D occasion shown in Figure 1(b), the mapping way is from a vertex of a square cell to the adjacent one along an edge of this square cell. For the 3D occasion, the mapping route for level one shown in Figure 1(c) is from a vertex to another according to the vertical number in the cube cell; the mapping manner for level two is filling the eight cube cells in the order of the numbers shown in Figure 1(d) by using the mapping way in one cube cell for level one. Finally, the phantom is setup by placing the scatterers with predefined density, strength, and position distributions into a cube with a preset size. Details for this mapping algorithm can be found in [26]. Due to the ground truth that aim parameters could be finely preset, which could be hardly reached in physical phantom, this scatterer model is attractive for evaluation of parameter characterization and fitting performance of HK distribution for ultrasound RF signals from the cross-tissue simulation.

### 2.3. Parameter Estimation

Given an observed data set *X*, the log-likelihood value *L*(*s*) is computed with the known global probability density function (PDF) *P* and unknown parameters *s* as [27]

(7) $$\begin{array}{c} L\left(s\right)=\text{ln}\prod_{x\in X}P\left(s\;|\;x\right). \end{array}$$

The maximum likelihood estimation is seeking the estimated values of parameters *s* when *L*(*s*) attains its maximum. For realizing this object, *s* must conform as

(8) $$\begin{array}{c} \frac{\partial }{\partial s}L\left(s\right)=0. \end{array}$$

However, it is difficult to compute the parameter *s* directly by solving the analytic solution from (8). For this reason, it is necessary to use a method of numerical calculation to find this parameter by exploring the optimized result within a certain range under the condition of the maximum log-likelihood value. The solutions to (8) are found numerically using the Newton-Raphson method [28]. For this purpose, define

(9) $$\begin{array}{c} F_{1}\left(x;{\overset{⌢}{s}}_{1}^{k},{\overset{⌢}{s}}_{2}^{k},\;\ldots ,\;{\overset{⌢}{s}}_{M}^{k}\right)=\frac{\partial L\left(s\right)}{\partial s_{1}}{\;|\;}_{s={\overset{⌢}{s}}^{k}}=0,\\ F_{3}\left(x;{\overset{⌢}{s}}_{1}^{k},{\overset{⌢}{s}}_{2}^{k},\;\ldots ,\;{\overset{⌢}{s}}_{M}^{k}\right)=\frac{\partial L\left(s\right)}{\partial s_{3}}{\;|\;}_{s={\overset{⌢}{s}}^{k}}=0,\\ F_{M}\left(x;{\overset{⌢}{s}}_{1}^{k},{\overset{⌢}{s}}_{2}^{k},\;\ldots ,{\overset{⌢}{s}}_{M}^{k}\right)=\frac{\partial L\left(s\right)}{\partial s_{M}}{\;|\;}_{s={\overset{⌢}{s}}^{k}}=0, \end{array}$$

where *s* = [*s*_1_, *s*_2_,…, *s*_*M*_] is the model parameter vector; $\overset{⌢}{s}=\left[{\overset{⌢}{s}}_{1},{\overset{⌢}{s}}_{2},\ldots ,{\overset{⌢}{s}}_{M}\right]$ is the estimated one at the *k*th iteration. The value of the parameter vector *s* at a given iteration is obtained as

(10) $$\begin{array}{c} {\overset{⌢}{s}}_{1}^{k+1}={\overset{⌢}{s}}_{1}^{k}-\frac{F_{1}\left(x;{\overset{⌢}{s}}_{1}^{k},{\overset{⌢}{s}}_{2}^{k},\;\ldots ,\;{\overset{⌢}{s}}_{M}^{k}\right)}{F_{1}^{\prime }\left(x;{\overset{⌢}{s}}_{1}^{k},{\overset{⌢}{s}}_{2}^{k},\;\ldots ,\;{\overset{⌢}{s}}_{M}^{k}\right)} \end{array}$$

(11) $$\begin{array}{c} {\overset{⌢}{s}}_{2}^{k+1}={\overset{⌢}{s}}_{2}^{k}-\frac{F_{2}\left(x;{\overset{⌢}{s}}_{1}^{k},{\overset{⌢}{s}}_{2}^{k},\;\ldots ,\;{\overset{⌢}{s}}_{M}^{k}\right)}{F_{2}^{\prime }\left(x;{\overset{⌢}{s}}_{1}^{k},{\overset{⌢}{s}}_{2}^{k},\;\ldots ,\;{\overset{⌢}{s}}_{M}^{k}\right)} \end{array}$$

(12) $$\begin{array}{c} {\overset{⌢}{s}}_{M}^{k+1}={\overset{⌢}{s}}_{M}^{k}-\frac{F_{M}\left(x;{\overset{⌢}{s}}_{1}^{k},{\overset{⌢}{s}}_{2}^{k},\;\ldots ,\;{\overset{⌢}{s}}_{M}^{k}\right)}{F_{M}^{\prime }\left(x;{\overset{⌢}{s}}_{1}^{k},{\overset{⌢}{s}}_{2}^{k},\;\ldots ,\;{\overset{⌢}{s}}_{M}^{k}\right)}. \end{array}$$

The value of *s*_1_ obtained from (10) is used as the initial value in (11), whereas the value of *s*_2_ found in (11) is used as the initial value in the subsequent equation and so on. Finally, the value of *s*_*M*_ found in (12) is used as the initial value of *s*_*M*_ in solving (10) in subsequent iterations. This iterative process will be continued until the following condition is satisfied:

(13) $$\begin{array}{c} \left|\overset{M}{\underset{i=1}{\sum }}{\overset{⌢}{s}}_{i}^{k+1}-{\overset{⌢}{s}}_{i}^{k}\right|\leq 1\times 10^{-8}. \end{array}$$

In the MLE processing, the PDF of the used statistical models should be calculated firstly. For the Rayleigh, Rician, and K distributions, the analytical expression defined as (1), (2), and (3) could be directly used to calculate the PDF. For HK distribution, its PDF expression defined as (4) is an integral form, from which its analytic primitive function is hard to be obtained. Thus, the numerical integration is used to compute this integral for the PDF by the function *quadgk* in the MATLAB platform, which returns the integral result using a high-order global-adaptive Gauss-Kronrod quadrature [29] with input parameters of integration range (0, inf), 1 × 10^−8^ of error tolerance and 20,000 of allowed maximum number of intervals.

## 3. Experiments

### 3.1. Experiments with Simulation Data

In the simulation study, echoed ultrasound RF signals and their corresponding envelope images are simulated by using the Field II library from a set of 3D scatterer phantoms based on gamma distributions with different values of shape and scale parameters, which control to implement continuously from the clustered to random to uniform scatterer distributions firstly. In the simulation, the scatterer phantom is set as a cube with 12 mm × 12 mm × 12 mm under the transducer surface of 20 mm with shape parameter values of 0.1, 1, 10, and 100, as well as density values of 5, 10, 50, and 100 scatterer/ *λ* ^3^, respectively. Acoustic parameters are set as the center frequency of 5 MHz, the sampling frequency of 100 MHz, sound speed of 1540 m/s, and wavelength of 3.08 × 10^−4^ m; the parameters for the linear array transducer are physical and active elements of 512 and 64, respectively, element width of 1.54 × 10^−4^ m, height of 0.005 m, fixed focal point of [0, 0, 0.03] m, respectively, and lines for envelope imaging of 20. The mean and variance for normal distributions of scatterer strength are 0 and 1, respectively. Then, the MLE for statistical histograms (SH) of the gray levels of the envelope speckle images is performed to obtain the values of the model parameters and log-likelihood. In order to evaluate the parameter meanings and goodness of fit for the HK distribution, the mean and standard deviation of these estimated values based on 30 realizations are compared with those by the optimally fitting models chosen from commonly used three single distributions.

### 3.2. Experiments with Human Subjects

The modeling performance of the homodyned K distribution for ultrasonic speckles from scatterers with varying spatial organizations is also accessed by B-mode images of common carotid arteries scanned from a small group of volunteers. The carotid artery is the arterial trunk on both sides of the head and neck. Due to its special anatomical structure, cardiovascular and cerebrovascular diseases such as atherosclerosis are usually initialized and developed from this arterial segment. Geometric and statistical information obtained by using ultrasound techniques from the intima-media, media, adventitia, blood flow, and surrounding tissues of CCA has important clinical significance for disease diagnosis [30]. It has been proved by histological studies [31] that the intima composed of the endothelium and subendothelial is the thinnest inner layer of the vessel wall. The adventitia is made up with the loose connective tissue, and the inoblast is the main cellular constituent of the loose connective tissue in the vessel wall. The media, which locates between the intima and adventitia, is composed of the elastic membrane with a little smooth muscle, whose reflection effect for ultrasound could be attenuated due to its location between the intima and adventitia. The blood in the lumen, which is one of the connective tissues, is mainly composed of the plasma and hemocyte, and the echo is mainly produced by the hemocyte. Thus, the intima, intima-media, adventitia, and blood in the lumen could be considered as a set of test tissue samples with different scatterer distributions and spatial organizations.

All clinical B-mode ultrasound images are scanned by a commercial ultrasound system (PHILIPS iU22, Philips Medical Systems, Andover, MA) equipped with a L12-5 linear array transducers. The imaging parameters are set as the grey level of 55%, the contrast level of 56%, the overall gain of 6 (the maximum scale is 12), and the time gain compensation from the near field to far field of −4, −3, −2, −1, 0, 1, 2, 3, and 4 dB. 30 B-mode ultrasound images of healthy CCA are collected, and the sections of the intima-media, media, adventitia, and lumen are manually segmented from CCA images delicately. The envelope data are estimated by a nonlinear mapping method [8] from the B-scan data in each section. The histogram and maximum likelihood estimation are computed from these estimated envelope data for comparison. The mean and standard deviation of estimated model parameter and likelihood values based on HK distribution are also compared with those by the optimally fitting models chosen from commonly used three single distributions.

All the above simulation and performance evaluation are conducted with software platforms of Windows® XP and MATLAB R2014b, under the hardware conditions of Intel Pentium Dual-Core CPU (E6500) 2.93 GHz and 4 GB memory.

## 4. The Results and Discussions

### 4.1. Results and Discussions with Simulation

**Figure 2:**
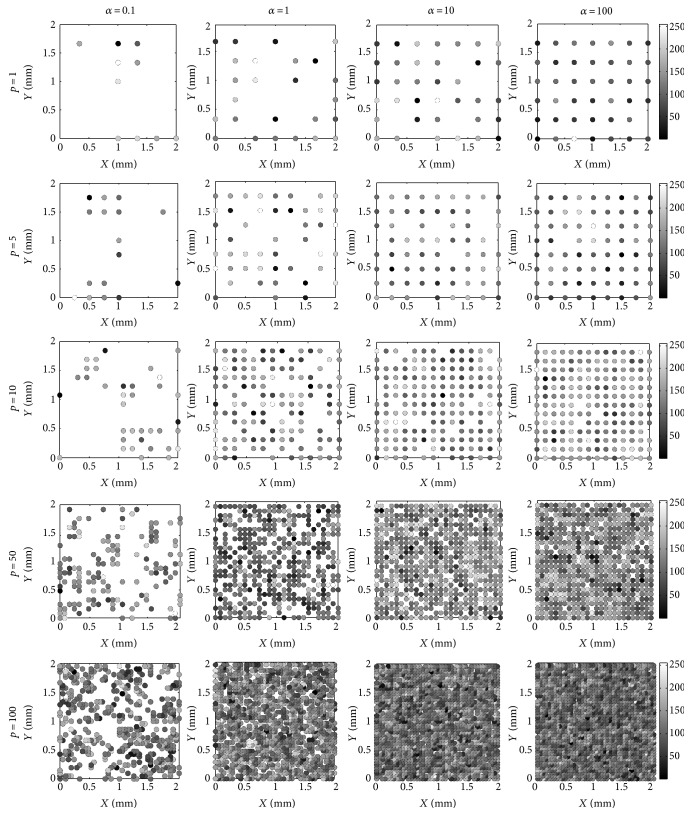
The scatterer phantoms with strength following normal distribution of mean 0 and variance 1. The image size is 2 mm × 2 mm.

In order to assess physical meanings of parameter and fitting performance of the HK distribution, 30 isotropous scatterer models with different spatial organizations are setup.  Figure 2 depicts the scatterer models with different shape *α* and density *ρ* values in the cases of the scatterer strength following normal distributions of mean 0 and variance 1. The scatterers are distributed as spreading dots, whose gray levels indicate the scatterer strength. In Figure 2, considered separately the influence of the shape parameter *α* on the scatterer distributions, it is observed that the scatterers are high clustered with a small value of *α* and the most tightly clustered for *α* = 0.01. However, the clustered distributions are becoming random and even approaching the uniformity along with the increasing density *ρ*. With the increasing value of *α*, the clustered scatterers are randomly and then uniformly spread in the space, notably the most uniform distributions for *α* = 100. Focused on the effect of the density parameter *ρ* on the scatterer distributions, it can be found that the scatterers cluster in the space for small *ρ* and become stochastic in manner and then in uniformity with the growing shape parameter *α*.

Therefore, both the shape parameter and scatterer density affect the scatterer distributions and effective scatterers in space. Lower values of shape and density parameters lead to more tightly clustered distributions, from which a few effective scatterers can be found. However, larger shape and density parameter values give more even distributions with more effective scatterers. It is known that the scatterer model reflects tissue characteristics by spatial distribution, which determines the speckle patterns in echoed signals.

**Figure 3:**
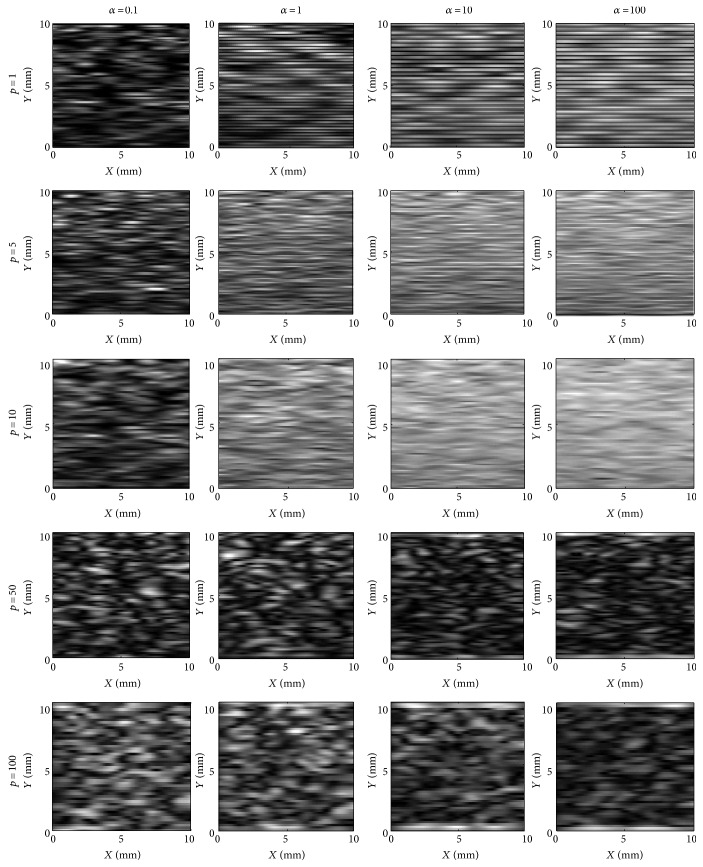
The simulated ultrasound envelope images. The image size is 12 mm × 12 mm.

Figure 3 shows the simulated ultrasound envelope images with different shape *α* and density *ρ* values corresponding to the scatterer models shown in Figure 2. In Figure 3, the variation of the shape parameter *α* influencing the results is checked firstly. With the low *α*, the clustered distributions in scatterer models (Figure 2) produce sporadic speckles without any deterministic component in images, particularly for *α* = 0.01, whereas the dispersive speckles without regular components are becoming denser randomly with the increase of density *ρ*. In the cases of increasing *α*, which represent that the scatterer distributions in the models turning gradually from clustering to regularity, the speckles in the images are becoming denser with more or less horizontal line patterns, which is particularly remarkable for *ρ* = 1. Especially for *α* = 100, the most regular speckle pattern can be found in the images. Secondly, considering the variation of the density *ρ*, the sporadic speckles, whose distributions are consistent with the those of the scatterer models shown in Figure 2, are also distributed in images when the *ρ* is small. The most clustered speckle distributions can be found for *ρ* = 1. The sporadic speckle distributions are changing to the densely random ones for the increasing shape parameter and the regular ones for the large-shape parameter. Thus, both the shape and effective density parameters affect the speckle distributions in the images. The shape parameter determines the regularity of speckle distribution for a certain degree of effective density.

The cases of *α* = 1, 10, and 100 should be drawn more attention. When *ρ* = 1, the speckle distributions present an increasing ordered horizontal-line pattern; for *ρ* = 5 and 10, the images show that the major speckle areas are highlighted high-reflection regions, indicating the well-organized periodic structure; to *ρ* = 50 and 100, high-dense and uniform scatterer distributions result in total reflection occurring to some extent in corresponding ultrasonic envelope images. The total reflection is a physical phenomenon that the incident and reflected waves are counteracted due to their opposition when the characteristic impedance of the incident medium is much greater than that of the reflective medium, and contrarily, the amplitude of the synthesis wave is double of that of the incident wave based on the in-phase [32]. Thus, in the images (for *α* = 1, 10, and 100 and *ρ* = 50 and 100), strong specular reflection appears in the belt zones of the top and bottom, and weak random reflection appears in the central region.

**Figure 4:**
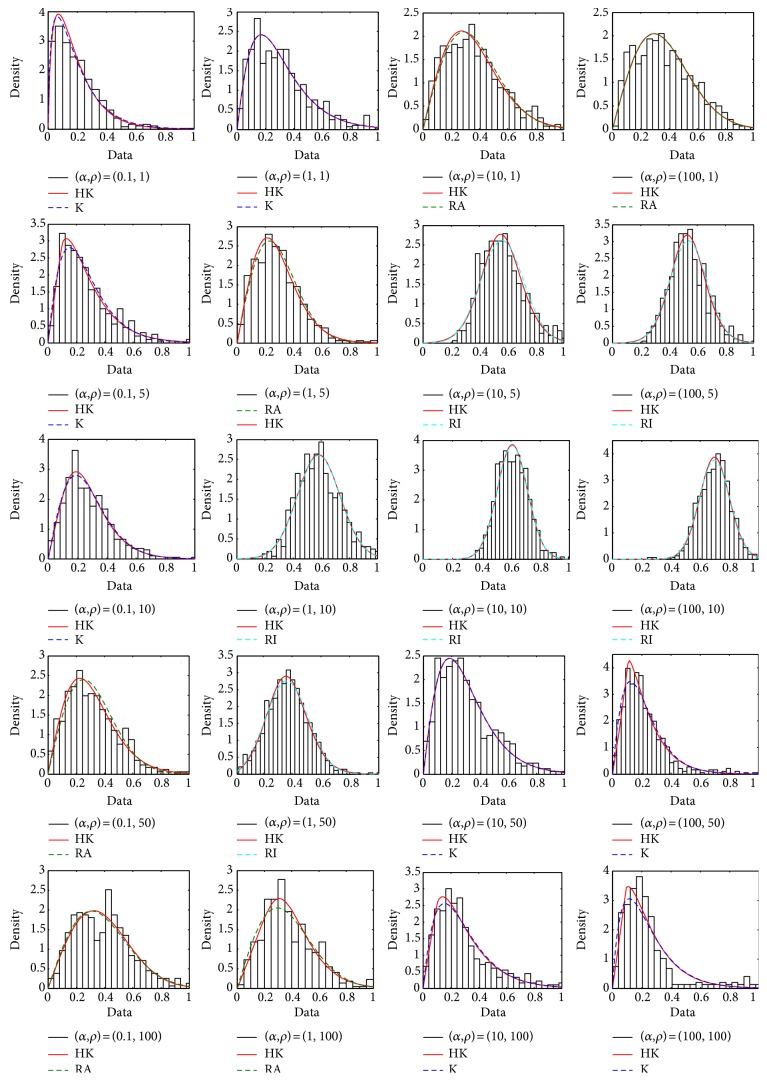
The statistical histograms and fitted curves for simulated ultrasound envelope images.

Figure 4 demonstrates the statistical histograms and fitted curves for simulated ultrasound envelope images in Figure 3. In these subfigures, the statistical histograms express the distributions of echoed envelope data, and the two fitted curves represent the estimated PDF waveforms of the HK (red line) and the model chosen optimally from the K (blue line), Rayleigh (green line), and Rician (turquoise line) under the MLE condition. In Figure 4, considered only the role of the shape parameter, when the *α* is small, the OKRR model for the echo envelope, which corresponds to the clustered scatterers (shown in Figure 2) and the sporadic speckle images (shown in Figure 3), is the K distribution. Especially, the case of *α* = 0.01 is the most typical. Moreover, the differences of goodness of fit between the K and HK distributions can be found. The goodness of fit of the HK distribution for the envelope data is better than that of the K distribution. With rises of shape parameter and density, the fitted distributions by the OKRR are turning into the Rayleigh. In these cases, Rayleigh and HK distributions for fitting the histograms of the given data are very close. With continuous increase of density or shape parameter, the fitted models by the OKRR are becoming the Rician distributions, whose goodness of fit is approximately equal to the HK distribution. The cases of *α* = 1, 10, and 100 should be noticed again. When *ρ* = 1, the dispersive histograms represent the envelope data from scatterers with low density and a little organized periodic structure (shown in Figure 2), as well as the speckle distributions with an ordered horizontal-line pattern (shown in Figure 3). In these cases, both the OKRR-based K or Rayleigh distributions and the HK distributions could conform well to the dispersive statistical histograms; for *ρ* = 5 and 10, the OKRR-based Rician distributions conform well to statistical histograms due to the more regular distributions in space for high-dense scatterers; for *ρ* = 50 and 100, which represent the very high regularity and density of scatterers, the OKRR-based K distributions rather than the Rician distributions are the preferable options for modeling the envelope data due to the appearance of the total reflection in images. Thus, the HK distribution could be a better model for the fitness with the ultrasonic speckle in envelope images echoed from very high regular and dense scatterer distributions.

**Figure 5:**
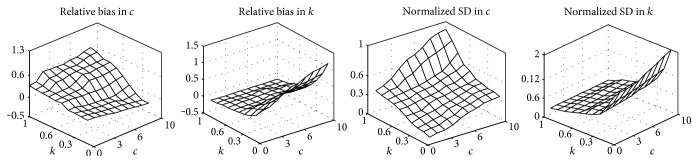
Relative bias and normalized standard deviation of parameters of HK distribution for random number.

In order to demonstrate the performance for the parameter estimation for HK distribution using the MLE algorithm, relative bias and normalized standard deviation (SD) of the values of the two parameters *c* and *k* = *ε*/*σ* [12, 13] with sets *c* ∈ {1, 2,…, 10} and *k* ∈ {0.1, 0.2,…, 1.0} for 1000 random numbers are computed and shown in Figure 5. Compared with the results of [12, 13], the algorithm based on MLE to estimate the parameters of HK distribution get smaller errors. However, the average time for one estimation takes 3–8 minutes, which is much longer than those methods of moments. More studies on the selection of initial values of parameters, numerical computation of PDF, and solution of the maximum likelihood equation are needed in the future for improving computational efficiency of the presented method.

In order to quantitatively assess the parameter characterization and goodness of fit, Table 1 lists the mean and standard deviation of the estimated parameters and maximum likelihood values of the fitted distributions with different shape *α* and density *ρ* values. To obtain a more reasonable comparison for distributions having different numbers of free parameters, the “best-fitting” value was calculated using the likelihood value based on minimized Schwarz's Bayes information criterion (BIC) [33]:

(14) $$\begin{array}{c} L=-2L_{l}+m\text{log }n, \end{array}$$

where *L*_*l*_ is the value of the maximum log-likelihood, as well as *n* and *m* are the numbers of data samples and parameters in the model, respectively.. The likelihood values based on BIC of the fitted distributions by the HK, OKRR-based K, Rayleigh, and Rician are denoted as *L*_HK_,  *L*_K_,  *L*_RA_, and *L*_RI_, respectively. The asterisk symbols ∗, ∗∗, ∗∗∗, and ∗∗∗∗ that are reflected on the tables indicate the sections of the fitted OKRR distributions by the models of K, Rayleigh, Rician, and K with total reflection, respectively. It is commonly known that the three parameters *ε*, *σ*, and *c* of the HK distribution have their independent physical implications, which separately express the coherent component, diffuse component, and clustering degree of scatterers in tissue, respectively.

It has been shown from the scatterers (Figure 2) and the corresponding envelope images (Figure 3) that the speckle distributions in images are clustered from high-variance (*α* < 1) and low-density scatterers (*ρ* ≤ 10). In the corresponding section ∗ (OKRR-based K distributions) of Table 1, the small density or shape parameters mean the low effective density or high clustered scatterers with little organized periodic structure. In these cases, the estimated *ε* ranged as 0.01–0.14 indicates existence of little coherent components and increases along with the increasing density; the values of *σ* are from 0.17 to 0.26, which are also increasing along with the increasing density; *c* is small for high clustering and increasing along with the increase of the shape parameter and density, indicating a change from clustered to random to regular speckle distributions in images. Secondly, the speckle distributions become random around *α* = 1 and more even with *α* > 1. Meanwhile, adequate speckle is forming when *ρ* > 10 in the envelope images. Thus, section ∗∗ (OKRR-based Rayleigh distributions) denotes the random scatterers with higher effective density and more or less well-organized periodic structure. In these cases, the ranges of the estimated *ε* in section ∗∗ are 0.01–0.36, which are larger than those in section ∗ overall. This means more deterministic components in the echoed envelope data in section ∗∗ due to their scatterers having more regular spatial distributions with a higher shape parameter *α*. The range of *σ* in section ∗∗ is 0.16–0.30, which is close to that (0.17–0.26) in section ∗. Parameter *c* ranged as 5.61–100 in section ∗∗ is larger than that ranged as 1.00–2.55 in section ∗, which indicates weaker clustering in section ∗∗. Thirdly, Figure 3 shows that the speckle distributions become regular when *α* ≥ 1. Meanwhile, adequate speckle is forming when *ρ* = 5 and 10 in the envelope images. In its corresponding section ∗∗∗ (OKRR-based Rician distributions), which denotes the high effective density scatterers with a certain well-organized periodic structure, the range of estimated *ε* is 0.52–0.69, much larger values than those in sections ∗ to ∗∗. This implies more deterministic components in envelope data in this section due to much more regular distributions of scatterers with a higher parameter *α* and adequate density. The range of *σ* in section ∗∗∗ in the table is 0.11–0.16, smaller values than those (0.16–0.30) in section ∗∗, signifying a less random degree for envelope data in this section. The larger parameter *c* ranged as 5.69–40.29 indicates strongly even speckle distributions in envelope data echoed from the specular scatterer distribution in section ∗∗∗. Finally, it should be noticed that the clear characterization of the HK parameters corresponding to scatterer spatial organizations in section ∗∗∗∗ could not be found owing to the total reflection in the echoed envelope images. In summary, the parameters of the HK distribution still present their respective physical meanings of independent contributions from the clustered, random, and regular components in the scatterer distributions under MLE criteria.

In order to evaluate the goodness of fit for HK distribution modeling the ultrasonic envelope data, the largest difference between the values of maximum likelihood based on BIC of the fitted distributions by the HK and OKRR are listed in all four sections in Table 1 for comparison. The largest differences exist between *L*_HK_ = 245 and *L*_K_ = 239 for (*α*,  *ρ*) = (0.1,  5) in section ∗, *L*_HK_ = 159 and *L*_RA_ = 157 for (*α*,  *ρ*) = (0.1,  50) in section ∗∗, *L*_HK_ = 232 and *L*_RI_ = 228 for (*α*,  *ρ*) = (10,  5) in section ∗∗∗, and *L*_HK_ = 449 and *L*_K_ = 421 for (*α*,  *ρ*) = (100,  50) in section ∗∗∗∗. It can be found that goodness of fit for the HK distribution is close to or slightly better than that for OKRR models for random or mildly clustered or mildly regular scatterer distributions. In this case, the maximum relative difference of MLE is 1.27%. However, the HK presents better goodness of fit for clustered scatterers or random or mildly regular with well-organized periodic structure with a maximum MLE difference of 6.23%.

For the HK distribution, the SNR of simulated data can be expressed as [11]

(15) $$\begin{array}{c} SNR=\frac{\varepsilon^{2}+2\sigma^{2}c}{2\sigma \sqrt{\varepsilon^{2}c+2\sigma^{2}c+c^{2}\sigma^{2}}}, \end{array}$$

where *ε*, *c*, and *σ* are the parameters of the HK distribution. Based on (15), the numerical values of the SNR of simulated data are presented in Table 2. The SNR increases with an increasing scale parameter *α* or density parameter *ρ* before the emergence of total reflection and decreases with increasing *α* or *ρ* when total reflection occurs.

In present study, we use the point scatterer to make the 3D scatterer distribution by the given scatterer density and shape parameter of the gamma distribution; then, the corresponding RF, envelope signals, and B-mode images are obtained. For the scatterer model presented in [12, 13], two schemes were considered. First, randomly located scatterers were placed in the phantom volume at spatial locations distributed according to a uniform distribution; second, a fixed density of randomly located scatterers combined with coherent scattering created by using periodically spaced scatterers in different regions in the phantom were considered. In present study, a 3D scatterer phantom based on gamma distributions is built to be implemented from the clustered to random to uniform scatterer distributions continuously. Compared with previous scatterer models, the advantage for the presented 3D scatterer phantom is more flexible and controllable to synthesize a varied ultrasonic data for assessment of HK distribution performance.

### 4.2. Results and Discussions with Human Subjects

**Figure 6:**
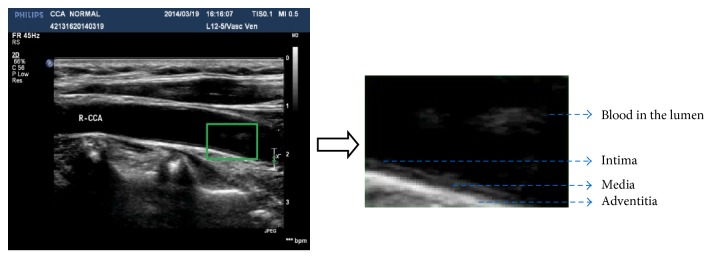
The ultrasound B-mode image scanned from a normal CCA (left) and the magnified region indicated by a green box for different tissue segmentation.

**Figure 7:**
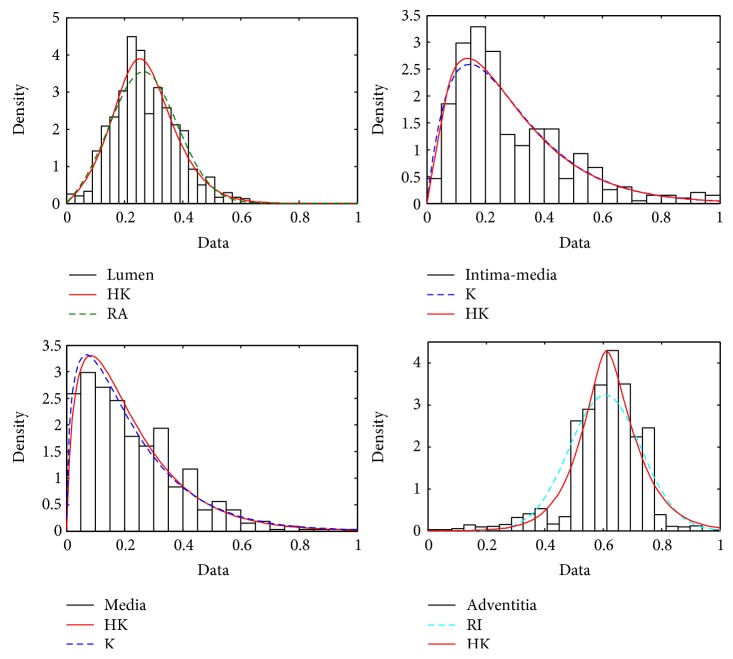
The statistic histograms of the envelope data and fitted curves for the lumen, intima-media, media, and adventitia.

30 B-mode ultrasound images of normal common carotid arteries of human subjects are scanned and recorded for validation using a L12-5 linear array transducer, which has 256 elements with a bandwidth of 5–12 MHz.  Figure 6 shows a B-mode ultrasound image scanned from a normal CCA (left) and the magnified region indicated by a green box for a different tissue segmentation. Their subimages are manually segmented delicately from CCA B-mode images and then converted into envelope data by a nonlinear mapping algorithm [8], from which the histogram and maximum likelihood estimation are computed for each kind of tissue data.  Figure 7 shows statistic histograms and fitted curves for the lumen, intima-media, media, and adventitia segmented from 300 envelope data converted from the image in Figure 6. It can be found that the echo envelopes of the blood, intima-media, media, and adventitia are OKRR-based Rayleigh, K (with a high coherent component), K (with a little coherent component), and Rician distributions. In general, the HK distribution conforms better with the envelope data than the OKRR distribution, especially for those speckle distributions with high coherent components.  Table 3 lists the mean and standard deviation of the estimated parameters and maximum likelihood values based on BIC from 30 test samples from 30 different images. It can be found that the goodness of fit compared with the OKRR results and estimated parameter values reflecting the tissue and speckle distribution characterization by using the HK distribution is accordant with the simulation results.

## 5. Conclusion

This paper presents an assessment of physical meanings of parameter and goodness of fit for homodyned K distribution modeling ultrasonic speckles from scatterer with wide-varying spatial organizations by using maximum likelihood estimation criteria. A set of 3D scatterer phantoms based on gamma distributions are built to implement from the clustered to random to uniform scatterer distributions continuously. The model parameters and maximum likelihood estimation are obtained by MLE from statistical histograms of the ultrasonic envelope data and then evaluated with a comparison with those of the optimally fitting models chosen from three single distributions, that is, the K, Rayleigh, and Rician distributions. The simulation results show that the parameters of the HK distribution still present their respective physical meanings of independent contributions in the scatterer distributions under MLE criteria. Moreover, the HK presents better goodness of fit with maximum relative MLE difference of 6.23% for random or clustered scatterers with well-organized periodic structure. Experiments based on ultrasonic B-mode images from common carotid arteries of human subjects validate the modeling performance of HK distribution for tissues with varying scatterer spatial organizations. It is concluded that the HK model for ultrasonic speckles is a better choice for characterizing tissue with a wide variety of spatial organizations based on the MLE, especially the emphasis on the goodness of fit for the tissue with well-organized deterministic components in practical applications. It may provide us more useful information for further applications by statistical analysis of the speckle properties in the ultrasonic images by the HK models.

## Figures and Tables

**Table 1:** The MSD of the estimated parameters and likelihood values based on BIC for simulated envelope data.

	*α* = 0.1		*α* = 1		*α* = 10		*α* = 100	
	HK	OKRR	HK	OKRR	HK	OKRR	HK	OKRR
*ρ* = 1	*L*_HK_ = 511 ± 18^∗^	*L*_**K**_ = 513 ± 16^∗^	*L*_HK_ = 220 ± 9^∗^	*L*_**K**_ = 220 ± 12^∗^	*L*_HK_ = 179 ± 7^∗∗^	*L*_RA_ = 179 ± 7^∗∗^	*L*_HK_ = 168 ± 12^∗∗^	*L*_RA_ = 168 ± 12^∗∗^
	*σ =* 0.17 ± 0.04^∗^	*m* = 0.87 ± 0.03^∗^	*σ =* 0.26 ± 0.02^∗^	*m* = 1.92 ± 0.15^∗^	*σ =* 0.29 ± 0.03^∗∗^	*d* = 0.29 ± 0.02^∗∗^	*σ =* 0.30 ± 0.04^∗∗^	*d* = 0.30 ± 0.04^∗∗^
	*c =* 1.00 ± 0.00^∗^	*a* = 7.48 ± 0.75^∗^	*c =* 1.92 ± 0.65^∗^	*a* = 7.50 ± 0.52^∗^	*c =* 12.84 ± 9.85^∗∗^		*c =* 100.00 ± 0.00^∗∗^	
	*ε* = 0.01 ± 0.00^∗^		*ε* = 0.01 ± 0.01^∗^		*ε* = 0.01 ± 0.01^∗∗^		*ε* = 0.01 ± 0.00^∗∗^	

								
*ρ* = 5	*L*_HK_ = 245 ± 57^∗^	*L*_**K**_ = 239 ± 64^∗^	*L*_HK_ = 240 ± 25^∗∗^	*L*_RA_ = 241 ± 23^∗∗^	*L*_HK_ = 232 ± 26^∗∗∗^	*L*_RI_ = 228 ± 32^∗∗∗^	*L*_HK_ = 298 ± 30^∗∗∗^	*L*_RI_ = 297 ± 30^∗∗∗^
	*σ =* 0.21 ± 0.03^∗^	*m* = 2.14 ± 0.23^∗^	*σ =* 0.16 ± 0.00^∗∗^	*d* = 0.31 ± 0.04^∗∗^	*σ =* 0.16 ± 0.00^∗∗∗^	*l* = 0.54 ± 0.06^∗∗∗^	*σ =* 0.13 ± 0.01^∗∗∗^	*l* = 0.52 ± 0.03^∗∗∗^
	*c =* 1.25 ± 0.32^∗^	*a* = 9.25 ± 0.32^∗^	*c =* 5.61 ± 2.68^∗∗^		*c =* 5.69 ± 3.54^∗∗∗^	*g* = 0.16 ± 0.03^∗∗∗^	*c =* 6.38 ± 2.64^∗∗∗^	*g* = 0.13 ± 0.08^∗∗∗^
	*ε* = 0.10 ± 0.05^∗^		*ε* = 0.36 ± 0.02^∗∗^		*ε* = 0.54 ± 0.02^∗∗∗^		*ε* = 0.52 ± 0.041^∗∗∗^	

								
*ρ* = 10	*L*_HK_ = 259 ± 38^∗^	*L*_**K**_ = 259 ± 40^∗^	*L*_HK_ = 268 ± 31^∗∗∗^	*L*_RI_ = 267 ± 31^∗∗∗^	*L*_HK_ = 409 ± 49^∗∗∗^	*L*_RI_ = 409 ± 49^∗∗∗^	*L*_HK_ = 411 ± 21^∗∗∗^	*L*_RI_ = 410 ± 20^∗∗∗^
	*σ =* 0.20 ± 0.01^∗^	*m* = 5.41 ± 0.27^∗^	*σ =* 0.14 ± 0.00^∗∗∗^	*l* = 0.53 ± 0.09^∗∗∗^	*σ =* 0.11 ± 0.03^∗∗∗^	*l* = 0.60 ± 0.00^∗∗∗^	*σ =* 0.11 ± 0.01^∗∗∗^	*l* = 0.69 ± 0.05^∗∗∗^
	*c =* 2.55 ± 0.54^∗^	*a* = 14.89 ± 1.02^∗^	*c =* 6.70 ± 4.52^∗∗∗^	*g* = 0.14 ± 0.05^∗∗∗^	*c =* 40.26 ± 31.81^∗∗∗^	*g* = 0.11 ± 0.02^∗∗∗^	*c =* 14.02 ± 3.57^∗∗∗^	*g* = 0.11 ± 0.01^∗∗∗^
	*ε* = 0.14 ± 0.04^∗^		*ε* = 0.53 ± 0.02^∗∗∗^		*ε* = 0.60 ± 0.02^∗∗∗^		*ε* = 0.69 ± 0.01^∗∗∗^	

								
*ρ* = 50	*L*_HK_ = 159 ± 17^∗∗^	*L*_RA_ = 157 ± 18^∗∗^	*L*_HK_ = 504 ± 29^∗∗∗∗^	*L*_RI_ = 501 ± 27^∗∗∗∗^	*L*_HK_ = 149 ± 20^∗∗∗∗^	*L*_**K**_ = 149 ± 18^∗∗∗∗^	*L*_HK_ = 449 ± 30^∗∗∗∗^	*L*_**K**_ = 421 ± 29^∗∗∗∗^
	*σ =* 0.25 ± 0.04^∗∗^	*d* = 0.25 ± 0.02^∗∗^	*σ =* 0.15 ± 0.02^∗∗∗∗^	*l* = 0.33 ± 0.05^∗∗∗∗^	*σ =* 0.26 ± 0.03^∗∗∗∗^	*m* = 2.52 ± 0.15^∗∗∗∗^	*σ =* 0.16 ± 0.01^∗∗∗∗^	*m* = 2.14 ± 0.03^∗∗∗∗^
	*c =* 6.82 ± 2.68^∗∗^		*c =* 6.82 ± 1.45^∗∗∗∗^	*g* = 0.15 ± 0.01^∗∗∗∗^	*c =* 2.51 ± 0.76^∗∗∗∗^	*a* = 8.77 ± 0.24^∗∗∗∗^	*c =* 1.10 ± 0.07^∗∗∗∗^	*a* = 11.46 ± 1.25^∗∗∗∗^
	*ε* = 0.01 ± 0.01^∗∗^		*ε* = 0.33 ± 0.01^∗∗∗∗^		*ε* = 0.01 ± 0.00^∗∗∗∗^		*ε* = 0.11 ± 0.05^∗∗∗∗^	

								
*ρ* = 100	*L*_HK_ = 96 ± 9^∗∗^	*L*_RA_ = 95 ± 6^∗∗^	*L*_HK_ = 138 ± 22^∗∗∗∗^	*L*_RA_ = 136 ± 24^∗∗∗∗^	*L*_HK_ = 169 ± 21^∗∗∗∗^	*L*_**K**_ = 168 ± 12^∗∗∗∗^	*L*_HK_ = 198 ± 32^∗∗∗∗^	*L*_**K**_ = 195 ± 24^∗∗∗∗^
	*σ =* 0.26 ± 0.03^∗∗^	*d* = 0.31 ± 0.09^∗∗^	*σ =* 0.23 ± 0.04^∗∗∗∗^	*d* = 0.30 ± 0.02^∗∗∗∗^	*σ =* 0.24 ± 0.02^∗∗∗∗^	*m* = 1.74 ± 0.03^∗∗∗∗^	*σ =* 0.20 ± 0.03^∗∗∗∗^	*m* = 1.37 ± 0.07^∗∗∗∗^
	*c =* 100 ± 0.00^∗∗^		*c =* 3.08 ± 1.42^∗∗∗∗^		*c =* 1.27 ± 0.28^∗∗∗∗^	*a* = 7.57 ± 0.83^∗∗∗∗^	*c =* 1.10 ± 0.12^∗∗∗∗^	*a* = 7.89 ± 0.75^∗∗∗∗^
	*ε* = 0.24 ± 0.09^∗∗^		*ε* = 0.27 ± 0.40^∗∗∗∗^		*ε* = 0.10 ± 0.04^∗∗∗∗^		*ε* = 0.09 ± 0.01^∗∗∗∗^	

^∗^Section I; ^∗∗^Section II; ^∗∗∗^Section III; ^∗∗∗∗^Section IV.

**Table 2:** The numerical values of the SNR of simulated data.

	*α* = 0.1	*α* = 1	*α* = 10	*α* = 100
*ρ* = 1	0.58	0.70	0.93	0.99
*ρ* = 5	0.65	0.97	1.09	1.15
*ρ* = 10	0.78	1.12	1.02	1.21
*ρ* = 50	0.88	0.96	0.75	0.67
*ρ* = 100	0.99	0.85	0.65	0.63

**Table 3:** The mean and standard deviation of the estimated parameters and likelihood values based on BIC from human subjects.

	HK	OKRR
Lumen	*L*_HK_ = 610 ± 25	*L*_RA_ = 605 ± 24
	*σ* = 0.12 ± 0.02	*d* = 0.12 ± 0.03
	*c* = 3.40 ± 0.12	
	*ε* = 0.24 ± 0.05	

Intima-media	*L*_HK_ = 115 ± 37	*L*_**K**_ = 114 ± 34
	*σ* = 0.24 ± 0.05	*m* = 1.57 ± 0.17
	*c* = 1.33 ± 0.24	*a* = 7.23 ± 0.75
	*ε* = 0.08 ± 0.01	

Media	*L*_HK_ = 350 ± 38	*L*_**K**_ = 351 ± 40
	*σ* = 0.20 ± 0.03	*m* = 0.83 ± 0.21
	*c* = 1.00 ± 0.28	*a* = 6.28 ± 0.68
	*ε* = 0.01 ± 0.01	

Adventitia	*L*_HK_ = 1154 ± 56	*L*_RI_ = 1088 ± 42
	*σ* = 0.12 ± 0.02	*l* = 0.59 ± 0.12
	*c* = 1.74 ± 0.45	*g* = 0.12 ± 0.04
	*ε* = 0.61 ± 0.04	
